# Understanding and Managing Metabolic Deficiencies Post Bariatric and Esophagectomy Surgeries: A Narrative Review of the Literature

**DOI:** 10.7759/cureus.60192

**Published:** 2024-05-13

**Authors:** Mina Daniel, Renad Al Dhib, Moises Mendoza, Saima N Tisekar, Ananya Reddy Cingireddy, Binish Essani, Ruchi Mahashabde, Sai Aditya Maddineni, Maria Kamel

**Affiliations:** 1 Internal Medicine, Memorial Hermann Health System, Houston, USA; 2 General Surgery, Mahsa University, Kuala Lumpur, MYS; 3 Internal Medicine, Universidad Centroccidental Lisandro Alvarado (UCLA), Barquisimeto, VEN; 4 Internal Medicine, University of Perpetual Help System DALTA, Las Piñas, PHL; 5 Internal Medicine, MountainView Regional Medical Center, Las Cruces, USA; 6 Internal Medicine, Jinnah Medical and Dental College, Karachi, PAK; 7 General Surgery, Gandhi Medical College, Bhopal, IND; 8 General Surgery, Avalon University School of Medicine, Willemstad, CUW; 9 Medicine, Columbus Central University School of Medicine, Ladyville, BLZ

**Keywords:** micronutrient deficiencies, bariatric surgery, malabsorption, esophageal cancer (ec), primary gastric carcinoma, esophagectomy, post-bariatric surgery, malnutrition risk

## Abstract

Gastrectomy and esophagectomy are the most performed surgeries in the treatment of both esophageal and gastric cancers. The type of esophagectomy depends on the type of malignancy, site of the tumor, criteria of resection, and field of resection. The three standard approaches to esophagectomy are the transhiatal approach, the left thoracoabdominal approach, and a three-stage procedure. The transhiatal approach involves abdominal and cervical incisions, while the left thoracoabdominal approach is a one-stage procedure that utilizes a single incision exposing the dissection field. The Ivor Lewis and McKeown esophagectomies are two-stage and three-stage surgeries that include laparotomy with right thoracotomy. Malabsorption often emerges as a significant postoperative complication following esophagectomy and gastrectomy surgeries. Malnutrition linked with these cancers has detrimental effects, including heightened rates of postoperative complications, elevated infection risks, delayed wound healing, reduced tolerance to treatment, diminished quality of life, and heightened mortality rates. Our narrative review summarizes and sheds light on solutions to treat malabsorption disorders and malnutrition after gastric bypass surgery. These solutions include methods such as adjustments, supplements, and treatment. Although more research is needed to confirm their effectiveness, these methods indicate potential for lowering the impact on patients' diets. By considering the beneficial implications of these effects and considering solutions, we aim to improve the management of these adverse effects, ultimately improving the overall health and postoperative outcomes of patients.

## Introduction and background

This article explores the issue of malabsorption and its effects, focusing on patients undergoing bariatric surgery and esophagectomy for gastric and esophageal carcinomas, and examining common complications and possible treatments. We investigate the causes of malabsorption after gastrectomy, highlighting reduced production of factors and lower gastric acid secretion as reasons. These disruptions hinder nutrient absorption, increasing the risk of malnutrition and related dangers. Furthermore, bariatric surgeries, such as Roux-en-Y gastric bypass (RYGB) and esophagectomy for cancer, bring added complexities that require monitoring and specific interventions to prevent deficiencies in nutrients. Despite advancements in surgery techniques and postoperative care, addressing malnutrition remains a challenge. Shortages of micronutrients persist, calling for strategies to meet the diverse needs of patients after surgery. In dealing with the challenges of malabsorption and malnutrition, in this scenario, our goal is straightforward: to improve the handling of these impacts, ultimately leading to improved results after surgery and a higher quality of life for individuals confronting the task of gastric and esophageal cancers.

## Review

Types of esophagectomies for esophageal cancer patients

Esophagectomy is mostly performed for malignant conditions, as benign conditions longing for esophagectomy are long-standing corrosive injuries and the formation of strictures. The type of esophagectomy depends upon the type of malignancy, choice of incision and approach, location of the tumor, criteria of resection, and field of resection. If the tumor is distal, esophagectomy has to be combined with partial or subtotal gastrectomy. If it is proximal, an additional pharyngectomy has to be performed to obtain local clearance [1].

There are three standard approaches: the transhiatal approach, the left thoracoabdominal, and a three-stage procedure. Firstly, the transhiatal approach was modified by Dr. Orringer in 1980 as an alternative for three-stage procedures and it spares the patient of the thoracoabdominal or pulmonary complications of thoracotomy [2]. The transhiatal approach involves abdominal incision and cervical incision. Through the abdominal incision, a full laparotomy is performed to check the evidence of any spillage or metastasis. The main aim is to create a gastric tube and its mobilization to the cervical esophagus and facilitate smooth gastroesophageal anastomosis. The blood supply to the gastric tube is mainly through the right gastric and gastroepiploic vessels. The left gastric artery and left gastric veins are ligated. The gastric tube, when prepared, is prepared as a conduit to be drawn up to the chest. When the stomach is prepared as a conduit, the duodenum is Kocherized. Now, the tube is passed through the esophageal hiatus to reach the esophagogastric junction. During these maneuvers, blunt dissection is done posterior to the esophagus, separating it from the prevertebral fascia and anteriorly avoiding any contact with the cardia to avoid arrhythmia [2].

Now, during cervical incision, the anterior border of the sternocleidomastoid is separated, dissection is done, and freed of all attachments, such as ligating the inferior and middle thyroid arteries and preserving the recurrent laryngeal nerves. Transection of the cervical esophagus is done after insertion of the nasogastric tube proximal to the line of transection. The stomach is moved up into the chest through the posterior mediastinum, and the highest part of the fundus is used for anastomosis. Anastomosis is created using a circular stapler or hand anastomosis. Alternate to the stomach, the colon or jejunum can also be used [2,3]. The overall morbidity was 50.8% and mortality was 8.7%. The most common complications were pulmonary at 29.9%, followed by gastrointestinal (14.9%), cardiovascular (10.4%), procedure-specific (7.8%), and infection (7.0%). The overall mortality was 8.7% [3,4].

The incidence of anastomotic leakage or stricture is around 10%, with mostly cervical leak, which needs to be drained, and insertion of feeding jejunostomy is needed for initiation of early enteral nutrition. If the leak is not repaired and endoscopic stenting fails, re-exploration is required [4].

The incidence of conduit ischemia is 10% and is repaired using serial dilatation [5].

Tracheal tears can occur intraoperatively during esophagectomy, followed by the placement of an endotracheal tube and ventilation [5].

Colonic redundancy is the most common late complication when using colonic interposition for esophageal reconstruction. This leads to mechanical dysfunction of the neo-conduit, causing disabling symptoms that may develop decades after the original surgery. When symptoms caused by food retention in the colonic loop occur, surgical correction may be necessary [6].

Secondly, thoracoabdominal esophagectomy is a suitable approach for tumors involving the distal esophagus, esophagogastric junction, and gastric cardia and involves distal gastrectomy. It is a one-stage procedure that utilizes a single incision exposing the dissection field. Post gastrectomy, the remaining stomach is used as a conduit, and anastomosis is done beneath the aortic arch; anastomosis can be hand-sewn or circularly stapled. If total gastrectomy is done, the reconstruction is with esophagojejunal anastomosis. A retrospective cohort study of all left thoracoabdominal esophagectomies was performed at a single specialist center over 11 years. Primary outcomes were in-hospital mortality, complications, resection margin involvement, and lymph node yield; secondary outcomes were one-year and five-year survival. A total of 211 esophagectomies were performed. In-hospital mortality was 5.7% (12/211) [7,8]. A total of 101 subjects (47.9%) had an uncomplicated recovery; 110 subjects (52.1%) developed at least one complication. There were 15 clinically significant anastomotic leaks (7.1%). Twenty-four subjects (11.4%) required emergency reoperation, the most common indication being anastomotic leakage. Complete tumor excision (R0 resection) was achieved in 151 of 211 cases (71.6%); the median lymph node yield was 24. One-year and five-year survival rates were 70% (147/211) and 21% (24/116), respectively. Left thoracoabdominal esophagectomy can combine a radical oncological resection with acceptably low mortality and morbidity. Respiratory complications are more because of the thoracic incision.

Ivor Lewis is a two-stage surgery including laparotomy with right thoracotomy [7,8].

Lastly, McKeown is a three-stage surgery that includes laparotomy and right thoracotomy with cervical components. Midline laparotomy is performed similarly to the transhiatal approach, along with the insertion of feeding jejunostomy. Right posterolateral thoracotomy is done for mobilization of the esophagus for anastomosis of the stomach conduit with the esophagus.

The advantages of the Ivor Lewis esophagectomy include excellent visualization of all parts of the operation, the ability to perform two-field lymphadenectomy, a lower leak rate, and a lower chance of injury to the recurrent laryngeal nerves [8]. The disadvantages include the pain associated with a right thoracotomy, the potential for higher respiratory complications, and increased toxicity if a leak occurs. If the anastomosis is performed high in the right chest, there is no huge gain in esophageal length resected for a “routine” cervical anastomosis. The cervical component is performed similarly to the transhiatal approach [8].

Mechanisms involved in malnutrition following esophagectomy

The primary motility disorders are delayed or accelerated gastric emptying and excessive reflux of upper intestinal contents into the stomach post-gastrointestinal reconstruction surgeries. These surgeries cause changes in upper gastrointestinal function due to the loss of duodenal or jejunal continuity and an impaired stomach remnant, which are known as postgastrectomy syndromes. These syndromes tend to be present in characteristic patterns. Diarrhea is a common symptom caused by rapid transit [9,10].

Dumping Syndrome

Dumping is a condition often resulting from the destruction or circumvention of the pyloric sphincter. Following pyloroplasty or distal gastrectomy, roughly 20% of patients experience noteworthy dumping symptoms. Even though the mechanism of the syndrome is not fully understood, it is commonly linked to emptying hyperosmolar chyme (especially carbohydrates) into the small intestine [10]. It is thought that the osmotic gradient causes fluid shifts from the vasculature to the lumen of the bowel, provoking a vasomotor response, and this has the potential to release certain vasoactive hormones, including serotonin and vasoactive intestinal polypeptide. Clinical presentation of postprandial dumping syndrome includes but is not limited to nausea, vomiting, bloating, abdominal pain and cramps, flushing, secretory diarrhea, tachycardia, sweating, dizziness, and syncope. It is essential to understand that most patients, irrespective of the method of reconstruction, may experience symptoms of dumping syndrome within 10 to 30 minutes after a meal. However, 25% of patients with dumping syndrome have a late onset, occurring one to three hours after eating. This results from a sharp drop in blood sugar due to an excessive insulin response to a high-carbohydrate meal. The main basis for the diagnosis of dumping syndrome is clinical criteria. The diagnosis has been supported by an upper GI series, a monitored glucose challenge, or gastric emptying tests [11,12]. Therefore, it is vital to closely monitor the symptoms and follow the recommended diet with dietary alterations, including a low carbohydrate meal, increased frequency and decreased size of meals, and fluid restriction around meals to slow gastric emptying. Octreotide may be considered in patients who are refractory to dietary modification [12]. Research conducted on 30 patients diagnosed with dumping syndrome demonstrated that monthly administration of octreotide long-acting repeatable (LAR), a long-acting formulation, and subcutaneous administration of octreotide three times a day both effectively decreased symptoms associated with dumping and improved quality of life. Patients expressed their preference for the monthly treatment [13].

Post-vagotomy Diarrhea

Diarrhea develops in approximately 30% of patients after truncal vagotomy [14]. The causality of this is not yet fully understood. Although the exact cause is unclear, it is possible that the rapid movement of unconjugated bile salts from the biliary tree denervated into the colon can cause diarrhea by stimulating secretion. Most patients with these symptoms spontaneously get better without requiring treatment. However, if the diarrhea persists, oral cholestyramine - a substance that binds bile salts - can be effective. In the past, a surgical option involved placing a 10 cm reversed jejunal loop in continuity with a 100 cm distal to the ligament of Treitz. This option is now rarely used [15,16].

Slow Transit

Post gastrectomy patients with slow transit often present with symptoms of nausea, vomiting (bilious or nonbilious), epigastric pain or bloating, and early satiety, leading to weight loss over time. The differential diagnosis of these symptoms includes gastric stasis, alkaline gastritis, and Roux stasis syndrome [16].

Gastric Stasis

Following gastric surgery, a tiny gastric remnant, postsurgical atony, or vagal denervation may cause decreased gastric emptying [16]. Symptoms consist of epigastric fullness with meals (early satiety), often followed by emesis of undigested food, abdominal pain, and weight loss. The evaluation of a patient suspected of post-gastrectomy gastric stasis syndrome begins with an upper gastrointestinal (GI) series with a small bowel series to define postsurgical anatomy and rule out mechanical obstruction. Upper endoscopy is also typically performed to rule out anastomotic strictures or marginal ulcers, which could cause or exacerbate gastric stasis. Upper endoscopy can also disimpact food bezoars commonly found in chronic gastric stasis patients. Impaired gastric emptying is best diagnosed quantitatively with a nuclear medicine solid food emptying test [17]. Symptoms attributed to a small gastric remnant will usually improve with small, frequent feedings and time to allow the remnant stomach to accommodate. Postoperative gastric atony may respond to prokinetic agents such as metoclopramide and erythromycin. Although there is some evidence that gastric pacing may improve the symptoms of primary gastroparesis, its widespread clinical use is yet to be achieved [17,18]. If dietary and medical therapies fail, re-operative gastrectomy may be required. Patients without prior partial gastrectomy should undergo subtotal (75%) gastrectomy; patients with prior partial gastrectomy should undergo near-total (95%) gastrectomy or total gastrectomy with esophagojejunostomy [17]. A Billroth II reconstruction with a Braun enteroenterostomy may be preferred to a Roux-en-Y reconstruction because of the potential Roux stasis syndrome associated with the latter [17].

Alkaline Gastritis

Operations that remove or bypass the pylorus lead to reflux of bile into the stomach. In most patients, there are no clinically severe sequelae. However, approximately 2% of patients develop alkaline reflux gastritis, a syndrome of persistent burning epigastric pain, and chronic nausea that is aggravated by meals. The diagnosis is made primarily by excluding other causes of symptoms, although endoscopy may reveal gastritis, and a technetium biliary scan can demonstrate excessive reflux of bile into the stomach [19]. Various medical therapies for alkaline gastritis have been reported, but none have proven particularly effective. Surgical therapies aim at separating the remnant stomach from duodenal content by interposing a loop of jejunum between them. Examples include Roux-en-Y reconstruction (with a 45 to 60 cm Roux loop), Henley loop (interposition of a 40 cm isoperistaltic jejunal loop between the gastric remnant and the duodenum), and Billroth II reconstruction with Braun enteroenterostomy (positioned 45 to 60 cm from the gastrojejunal anastomosis). The reoperative procedure is chosen based on a patient's existing anatomy and how much remnant stomach is left [20].

Roux Stasis Syndrome

After a sub-total or total gastrectomy, Roux-en-Y reconstruction is performed as the main reconstruction. In patients with intractable dumping syndrome, severe alkaline gastritis, and afferent loop syndrome patients are also treated with it prior to surgery [20]. Following Roux-en-Y reconstruction, a small percentage of patients develop Roux stasis syndrome. Patients will have vomiting, epigastric pain, and weight loss. Disordered motility of the Roux loop with net propulsive activity toward, rather than away from, the stomach is hypothesized to be the cause. When evaluating patients suspected of having this syndrome, upper GI series, upper endoscopy, and nuclear medicine stomach emptying study should be performed [20]. Prokinetic agents such as metoclopramide and erythromycin are used in the medical treatment of Roux stasis syndrome. Surgical treatment involves resecting the departing Roux loop and replacing it with a new Roux-en-Y repair in cases where medicinal therapy is unsuccessful. Additional resection of the remaining stomach (near-total or 95% gastrectomy) is also performed to prevent recurrence. To prevent this syndrome, alternative reconstructive techniques like Billroth II reconstruction with or without Braun enteroenterostomy should be used. This is because patients with a generous (over 50%) gastric remnant and those who have undergone truncal vagotomy are more likely to experience this condition [21].

Gallstones

Cholestasis, which is brought on by decreased gallbladder contraction from a variety of factors, including weight loss, vagotomy, lymph node dissection in the hepatogastric ligament, and non-physiologic reconstruction, can result in gallstones following gastrectomy for cancer [22]. The effects of acute oral erythromycin on gallbladder motility and upper GI symptoms in individuals who had undergone gastrectomy, both with and without gallstones, were assessed in a randomized, placebo-controlled ultrasonographic trial. In one study, using ursodeoxycholic acid daily decreased the percentage of patients who developed gallstones within a year after having a gastrectomy (5.3% in the 300 mg and 4.3% in the 600 mg groups, respectively), in contrast to 16.7% for the placebo [23].

Types of gastrectomies for gastric carcinoma patients

In recent years, there has been a concerning increase in cases of gastric carcinoma, a particularly aggressive form of stomach cancer. Consequently, there has been a rise in the number of gastrectomies performed, which involve either partial or complete removal of the stomach. While gastrectomies are often effective in stopping the progression of gastric carcinoma, they are not without their drawbacks. Due to substantial changes made to the structure and function of the digestive system after surgery, one major concern is the possibility of malnourishment and malabsorption. With four different forms of gastrectomies performed to treat gastric cancer, our research attempts to present a comprehensive evaluation of the malabsorption and malnourishment that follow these procedures. We will also look at how these techniques affect the care of the patient after surgery and consider possible countermeasures for these negative outcomes [24].

While gastrectomies are efficient in stopping the growth of gastric cancer, they come with special difficulties. Because the structure and function of the digestive system are altered after surgery, the body’s capacity to effectively digest and absorb nutrients is hampered, which frequently results in malabsorption and malnourishment. Malnourishment and malabsorption are common consequences following gastric surgery. The altered structure combined with reduced stomach capacity might hinder the absorption and processing of nutrients, leading to dietary deficits and malnourishment [24].

Understanding the various forms of gasterectomies used to treat gastric cancer is essential to understanding the complexities of postoperative malabsorption and malnourishment [24].

The Four Main Methods of Gastrectomy

Subtotal gastrectomy: With this procedure, a part of the stomach is surgically removed to create a smaller reservoir for food storage and digestion. It is a widely used technique that is typically used when malignant tumors are found in the lower portion of the stomach [24].

Total gastrectomy: With this procedure, the stomach is removed entirely, leaving the esophagus and small intestine connected directly. Usually, it is saved for more severe cases of gastric cancer or when there are several tumors dispersed throughout the stomach [24].

Proximal gastrectomy: This procedure involves the removal of the upper segment of the stomach adjacent to the esophagus. Subsequently, the lower section of the stomach is attached to the esophagus. This technique is commonly utilized for tumors located near the gastroesophageal junction [25].

Distal gastrectomy: In this method, the lower portion of the stomach is removed, while the upper section is preserved. The remaining stomach is then connected to the small intestine. Typically chosen for tumors located in the middle or lower part of the stomach, this technique aims to address localized cancerous lesions while preserving a portion of the stomach's function [26]. The different gastrectomy methods can lead to varying degrees of malabsorption and malnourishment due to the altered digestive physiology they induce. Understanding the implications of each technique is crucial for delivering optimal postoperative care to patients and minimizing the risk of nutritional deficiencies [26].

In the subsequent section of this paper, we will delve into the specifics of malabsorption and malnourishment following gastrectomy procedures.

Prevalence and characteristics of malabsorption

Following gastrectomy surgeries, frequently performed to combat gastric carcinoma, malabsorption often emerges as a significant postoperative complication. This impediment to nutrient absorption from the alimentary canal can precipitate malnourishment and its associated consequences. The prevalence of malabsorption varies, contingent upon the type of gastrectomy undergone. Research indicates that a substantial proportion, roughly 40%, of patients undergoing total gastrectomy encounter malabsorption [27].

The specific technique utilized in the gastrectomy can also be a determinant of the extent of malabsorption. The primary instigator of malabsorption is the cessation of the production of intrinsic factors in the stomach. This essential component aids in the absorption of vitamin B12, and its deficiency can lead to pernicious anemia. Additionally, reduced gastric acid secretion post gastrectomy hinders the activation of digestive enzymes and restricts the absorption of various nutrients. The consequences of malabsorption and malnourishment after gastrectomy are profound and can profoundly affect a patient's overall health and recovery post surgery. Malnourished patients face heightened susceptibility to various complications, including infections, delayed wound healing, and impaired organ functions [28].

Strategies to Minimize the Impact of Malabsorption and Malnourishment

It is critical to treat malabsorption and malnourishment in people who have had gastric surgeries. If they do not, it could seriously harm their general health and recuperation after surgery. Many tactics can be used to reduce side effects and encourage appropriate diet to maximize their nutritional condition and improve postoperative results. The first line of defense consists of dietary adjustments. To make sure they are getting enough nutrients, patients should receive individualized dietary guidance. They might be instructed to emphasize nutrient-rich foods, eat smaller, more often meals, and stay away from items that are difficult to digest. Another important tactic is to use supplements to manage vitamin deficits. For example, to compensate for the shortages brought on by a decrease in intrinsic factor production, patients would require vitamin B12 injections or oral supplements [29].

Nutritional absorption and digestion can be significantly aided by pharmacological therapies as well. Giving pancreatic enzymes or bile acid-binding agents can help with nutrition utilization and reduce malabsorption symptoms. It is imperative to acknowledge that the efficacy of these tactics may differ depending on the specific type of gastrectomy executed and patient-specific variables. To determine the effectiveness of these therapies and how best to execute them, more research is required [30].

Metabolic deficiencies following restricted procedures

The digestive tract serves as the first line of defense for the nutrients we ingest, aiding in their absorption, digestion, and post-meal metabolism. The movement of food through the tract, which includes processes like emptying the stomach and the motion of the small intestine, controls feelings of fullness, calorie intake, nutrient absorption rates signaling by gut hormones, and the composition of gut bacteria. These factors affect how well blood sugar is controlled and how sensitive the body is to insulin. Changes in how food moves through the system could play a part in causing metabolic disorders. Issues such as stomach expansion nutrients reaching the small intestine and hormone release within the gut are thought to be involved in this process. Surgical procedures like sleeve gastrectomy (SG) change how our digestive system is structured and lead to metabolic alterations. SG involves removing part of the stomach and creating a tube for nutrients to pass through. Research on how these surgeries affect how our bodies handle carbohydrates after eating shows changes in blood sugar levels and insulin response [31].

Comparing laparoscopic sleeve gastrectomy (LSG) and RYGB, research indicates that LSG carries a risk of deficiencies compared to RYGB, which has a higher risk due to its more complex changes to the digestive system. While both procedures can result in weight loss outcomes, they may also lead to micronutrient deficiencies. Laparoscopic adjustable gastric banding falls under surgeries, keeping the digestive tract intact and connecting nutritional issues with food intake quantity and variety. Initially, after surgery, food intake is restricted, potentially causing consumption. Vitamins B1 and B12 deficiency may lead to serious complications like beriberi if not addressed quickly. Early diagnosis and treatment of thiamine deficiency are necessary [32].

Vitamin B12 deficiency, which has been reported in up to 26% of cases, is linked to decreased production of gastric acid and intrinsic factors following LSG surgery [32]. Neurological complications often occur concurrently with challenges like ulcers or sleeve stenosis. Long-term studies show that many patients require supplementation after LSG for iron, zinc, calcium, and cholecalciferol. Five years after the surgery, there are still instances of low serum ferritin levels due to changes in diet and reduced stomach acid post surgery. Checking serum ferritin levels is crucial for diagnosing iron deficiency. Iron therapy may be required for effective treatment [32].

The growing popularity of SG is mainly due to its nature and lower risk of complications compared to RYGB, especially regarding nutritional deficiencies. However, the exact effects of SG on calcium metabolism and bone health are not fully understood. The undisturbed intestinal tract after surgery indicates calcium absorption factors like existing vitamin D deficiencies, decreased stomach acidity post surgery, and dietary changes following weight loss could potentially affect calcium absorption. Nevertheless, the combined impacts of these factors on vitamin levels and their direct impact on calcium absorption capacity in SG patients have not been extensively studied, highlighting the need for exploration in this field. There is a belief that factors after surgery, such as changes in stomach acidity and hormones as adjustments in diet due to weight loss, might hinder calcium absorption. However, this theory has not yet been tested. While the surgery does not seem to impact the intestine's shifts in hormones and changes, diet following the operation could potentially reduce calcium absorption [32,33].

Both SG and RYGY avoid the fundus, which has specialized cells for digestion, like cells that make stomach acid (hydrochloric acid, HCl) and chief cells that release pepsin for breaking down proteins. This means that after surgery, digestion is compromised because these cells are missing in the fundus, affecting processes like calcium solubilization on stomach acid. The absorption of magnesium, crucial for bone health, is also impacted as it needs HCl for solubilization and might be affected by bypassing cells. Magnesium is mainly absorbed at the end of the intestine and colon through transient receptor potential melastatin type 6 (TRPM6). Magnesium is essential for bone health as it makes up 60% of bone magnesium and acts as an enzyme cofactor in regulating calcium metabolism. Besides its role in bone structure, magnesium is vital for functions such as adenosine triphosphate (ATP) production and enzyme activities involved in lipid, protein, and nucleic acid synthesis [33].

A study looking back at 1793 patients, with a majority being females (74%), who underwent LSG showed weight loss and changes in indicators over a span of five years. A significant drop in body weight was observed around 18 months after the LSG procedure. Despite the weight loss, there were health outcomes. Vitamin B1 levels notably decreased between three and five years post surgery and stayed within ranges. On the other hand, vitamins B6 and B12 initially rose at six months post operation before declining and not returning to surgery levels. Similarly, vitamin D levels improved from levels before surgery but remained insufficient throughout the study period. Markers like albumin, transferrin, folate, ferritin, iron, and vitamin B2 remained relatively stable during the five-year follow-up period with no deviations from surgical values. This study emphasizes the nature of changes following LSG surgery, with certain vitamins showing fluctuations over time. Although some vitamins initially saw improvements, after surgery, they later remained inadequate [34].

A recent research study found that many individuals undergoing LSG experienced an occurrence of vitamin deficiencies in vitamin B12, folate, iron, and vitamin D during the first year after the surgery. A significant number of patients developed vitamin B12 deficiency due to fundus resection during LSG, leading to the need for intramuscular supplementation to maintain levels and prevent complications like pernicious anemia and neuropathic pain. Folate deficiencies were less frequent but still notable because folate intake decreased post surgery, highlighting the importance of supplementation. Iron deficiencies leading to anemia affected a part of patients, stressing the importance of iron supplementation for prevention. Vitamin D deficiency was also common after LSG due to reduced bioavailability or obesity-related factors contributing to calcium and vitamin D inadequacies. These deficiencies can lead to metabolic bone diseases, underscoring the significance of detection and treatment. The study suggests evaluating status before surgery, promptly addressing any deficiencies, and providing supplementation and monitoring post surgery to minimize the negative impacts of micronutrient deficits following LSG [35].

The research emphasizes that many patients who undergo surgeries for weight loss commonly lack essential micronutrients and proteins. After the surgery, they are given supplements. They are advised to continue taking them indefinitely. Although it is not mandatory to correct these deficiencies before the surgery, the treatment provided afterward is considered adequate for managing and sustaining levels of nutrients, which is in line with standard practice. However, it is acknowledged that these surgeries could worsen existing deficiencies, leading to the need for both baseline and post-surgery supplementation, as recommended by research studies. It is recommended that assessments be conducted due to the increased risk of developing deficiencies after surgery. This underscores the importance of addressing deficiencies to improve outcomes and prevent new deficiencies from occurring [36].

Both RYGB and LSG are known to result in weight loss, although the debate continues regarding which procedure is more effective. Some studies suggest that RYGB may lead to better weight loss outcomes. A recent meta-analysis found no differences between the two surgeries in terms of weight loss. Both operations can impact calcium levels due to changes in bone structure following surgery. The research also noted an incidence of anemia often linked to deficiencies in iron and vitamin B12. Reduced production of factors after LSG could contribute to B12 absorption issues, while bacterial overgrowth in the intestine following RYGB might be a factor in B12 deficiencies. It is recommended that vitamin B12 be administered sublingually for supplementation for patients with coagulation problems or concurrent vitamin K deficiency. Iron deficiency, commonly seen in patients prior to surgery due to inflammation, may worsen following procedures [36].

Metabolic deficiencies following malabsorptive procedures

A few procedures regarding malabsorption have been recognized by the International Federation for the Surgery of Obesity (IFSO). These procedures include RYGB, one anastomosis gastric bypass (OAGB), single anastomosis duodeno-ileal bypass + sleeve gastrectomy (SADI-S), biliopancreatic diversion (BPD), and the biliopancreatic diversion with duodenal switch (BPD-DS). However, both BPD and BPD-DS account for the least used procedures as primary options [37]. They are the most classic procedures that are desired to achieve weight loss after RYGB or gastric sleeve failure, as well as the most common ones that lead to strong malabsorption [38].

Trace metals like iron, calcium, zinc, copper, selenium, and vitamins such as vitamins A, B9, B12, D, E, and K have come to be recognized as an indisputable late consequence after malabsorptive or mixed operations, which may lead to serious deficiency-related diseases [39]. In recent years, the American Society of Hematology reported that people who have had bariatric procedures are most at risk for anemia, mostly because of iron deficiency, with a secondary reason for vitamin B12 insufficiency. The malabsorptive process also influences the degree of iron deficiency. It is highest in individuals with BPD-DS (13-62%). For vitamin B12 deficiency, it is highest in patients who undergo BPD-DS or RYGB. Folate acid deficiency can manifest as macrocytic anemia, thrombocytopenia, leucopenia, or glossitis, which is primarily aggravated by vitamin B12 deficiency. Approximately 18% of individuals experience thiamine (vitamin B1) deficiency, which is linked to a variety of symptoms related to different organs [40,41].

Malabsorption in the jejunum and distal ileum causes malabsorption of fat-soluble vitamins, which includes vitamins A, E, K, and D. A total of 69% of patients experience vitamin A insufficiency four years after surgery. After two to four years following surgery, primarily in BPD-DS patients, the incidence of vitamin E deficiency ranges from 2% to 5%. The diagnosis of vitamin K insufficiency is only made by testing. No patient exhibits clinical signs of deficiency like early bruising, prolonged bleeding, and hemorrhage. Vitamin D deficiency occurs before or in the early stages after the surgery. This results from decreased vitamin D intake, decreased sun exposure, and storage in adipose tissue. A minimum dose of 3,000 IU per day is advised for prophylaxis before surgery and high doses after the surgery. Calcium deficiency is usually seen due to hypersensitivity against dairy products after surgery [41,42].

Preoperative screening of zinc is required before any malabsorptive procedures. An annual screening is required after RYGB or BPD-DS procedures. Zinc deficiency is seen to be highest in patients after BPD-DS. Like zinc, patients who follow recommended dietary supplementation have a very low chance of experiencing symptomatic copper deficiency following RYGB. Magnesium deficiency is one of the factors that contribute to the development of osteoporosis, but unlike zinc and copper, no screening or monitoring is required. Among the most serious issues with micronutrients, most commonly after BPD-DS, is protein malnutrition. Patients are advised to monitor their protein intake daily, and a daily protein intake of 60-120 g is recommended [38,43,44].

Impact on micronutrients

The process of breaking down food to extract nutrients for the body takes place in the intestine, particularly in the upper jejunum. Bariatric surgeries like RYGB and cases of esophageal and stomach cancers where esophagectomy and gastrectomy are performed can alter this process, potentially leading to deficiencies in trace metals. To address these deficiencies, patients post surgery may need supplements along with multivitamins. Deficient nutrients include iron, vitamin B12, vitamin D, folic acid, zinc, magnesium, vitamin B1, and certain minerals. Patients are closely monitored for malnutrition risks after surgery, focusing on protein levels, vitamins such as B12 and D, and calcium. Vitamin and mineral deficiency can have long-term health consequences post surgery, underscoring the importance of awareness and monitoring [38,43,44].

Surgery is used to treat stomach cancer, with esophagectomy and gastrectomy being two performed procedures. Thanks to advancements in techniques and postoperative care, the chances of removing tumors and achieving better results have increased. However, a significant challenge remains in managing malnutrition following surgery, which can result in weight loss, muscle depletion, and a lack of nutrients. Patients who have undergone gastrectomy or esophagectomy often face deficiencies in micronutrients like iron and vitamins A, B1, B12, D, and E due to changes in the body structure. Unfortunately, these deficiencies are often not adequately addressed, highlighting the need for management strategies. Advancements in both pre- and postoperative care after esophagectomy, along with the development of less invasive techniques, have lowered the risks of complications. However, assessing long-term quality of life has become a factor in determining success. The type of procedure chosen depends on factors such as tumor location and size as well as the overall health condition of the patient. Similarly, patients experience the same complications after gastrectomy, which is an option used to treat stomach cancer by removing part or all of the stomach [38,43,44].

The reconstruction method, often involving a Roux en Y anastomosis, may lead to malabsorption, to what is seen in gastric bypass surgeries. This malabsorption can result from defects in absorption of nutrients, changes in stomach acid production, alterations in diet, or decreased appetite and food intake. The impact of deficiencies following these surgeries can significantly impact the quality of life and overall outlook for patients. Timely detection and treatment of deficiencies like iron and vitamin supplements are essential for enhancing long-term outcomes and minimizing health risks. Additionally, closely monitoring patients' well-being and eating patterns is crucial for preventing and addressing complications related to malnutrition [45,46].

After undergoing esophagectomy surgery, many patients may experience deficiencies around six months post operation, as suggested by research. The most commonly affected micronutrients are vitamin B12, vitamin D, folate, and iron levels, although studies have not absolutely determined their occurrence. Changes in the patient's anatomy post surgery could be a contributing factor to these deficits. Managing micronutrients after esophagectomy differs from gastrectomy with Roux en Y procedures, as there is no established approach for monitoring and replenishing nutrients in esophagectomy patients. It is recommended that a team that includes a nutrition specialist be established to minimize the risk of micronutrient deficiencies following esophagectomy or partial gastrectomy procedures. This team could oversee care and conduct checks on vitamin levels at intervals ranging from one month to five years. Such an approach would be crucial in identifying any emerging deficiencies over time [44,47].

Iron

For individuals undergoing weight loss surgery, dealing with anemia and iron levels can be quite challenging both before and after the procedure. Studies indicate that these issues are more prevalent in this group. The decrease in iron levels post surgery can be attributed to factors such as the impact on iron levels, which varies depending on the type of surgery performed. Certain procedures, such as ring gastroplasty and adjustable silicone gastric banding, are uncommon to cause iron issues, but there is still a possibility of affecting iron levels. Following SG, initial concerns about iron deficiency are usually minimal. Still, there is a risk of developing iron deficiency anemia as a long-term complication with mini gastric bypass surgeries. Despite some surgeries being considered safe and efficient, like one anastomosis bypass, they may still lead to iron deficiency anemia, underscoring the challenges faced on the path to improved well-being [44,47].

After having RYGB surgery, patients may experience a drop in hemoglobin and ferritin levels despite an increase in serum iron levels after the procedure. On the other hand, individuals who undergo LSG may encounter fluctuations in their iron levels after the surgery. However, some patients might still observe normal hemoglobin and hematocrit levels. While there is no difference in how the body absorbs types of iron between SG and Roux en Y bypass, it is advisable for patients to take iron supplements to prevent deficiencies. Iron deficiency remains a concern after weight loss surgery. It is important to monitor and maintain mineral and multivitamin intake. Several factors, such as reduced efficiency in digesting meat, decreased stomach acid production, and changes in digestion processes, can lead to decreased iron levels post surgery. Specific demographic groups like menstruating women, pregnant women, and teenagers are more susceptible to developing iron deficiency and anemia following surgery. Diagnosing iron deficiency after weight loss procedures can be challenging due to causes and symptoms that overlap with nutrient deficiencies [44,47].

Healthcare workers often recommend conducting tests such as iron binding capacity or serum transferrin receptor levels to ensure the accuracy of the results. This is important because individuals who are overweight may show elevated ferritin levels due to inflammation. Additionally, there may be alterations in the sizes of their blood cells. Typically, physicians assess ferritin levels in the bloodstream with serum iron levels, transferrin levels, and total iron binding capacity measurements for diagnosis [48].

Patients may experience low iron levels after stomach resection, leading to iron deficiency anemia. This is especially common after partial or total stomach removal, as most surgeries bypass the upper intestine, which is important for absorbing iron. Additionally, the faster movement of food through the digestive tract post surgery reduces the time for iron absorption and decreased gastric acid in the intestine makes it difficult to transfer iron into a form that can be absorbed efficiently. These physical changes and a decrease in consuming iron-rich foods impact iron levels after stomach surgery. Treatment options are variable depending on the severity of the condition but often require taking iron supplements orally and sometimes intravenously in more severe cases. However, oral supplements may not always be effective in normalizing ferritin levels, emphasizing the importance of consistent supplementation [38,47].

Vitamin B12

Disturbances in the absorption process can occur after surgeries like bypass or resection, leading to deficiencies in vitamin B12. This process relies on stomach-generated factors and specific transporters in the ileum. Malabsorptive procedures switch bile and lipase away from food, affecting the absorption of fat-soluble vitamins and potentially causing deficiencies. While megaloblastic anemia is common following bypass, there is also a risk of long-term depletion of vitamin B12 reserves, necessitating oral supplementation for prevention. It is important to highlight the connection between the absorption of vitamin B12 and hydrochloric acid in the stomach since both are crucial for breaking down food and aiding vitamin B12 absorption in the terminal ileum [38,47].

About one-third or more patients might face vitamin B12 deficiency following RYGB surgery and total gastrectomy. This insufficiency is linked to a decrease in gastric acid, which plays a role in breaking down vitamin B12 from protein. Consequently, this shortfall could result in anemia that requires lifelong management with oral or injectable supplements. Signs of vitamin B12 deficiency typically show up two years or more post surgery, as the body can store B12 for about 12 to 18 months. Thus, ongoing monitoring through lab tests is crucial. Generally, possible explanations for the deficiency involve reduced intake of vitamin B12 in the diet due to changes affecting satiety, digestion of vitamin B12 from food due to decreased acid secretion, shortened exposure time to this acid in the conduit, and diminished production of intrinsic factors [45,47].

Vitamin B1

The onset of beriberi occurs when the body lacks thiamine, an important nutrient for glucose metabolism, oxygen delivery, and brain signaling. This deficiency can result in symptoms affecting different areas of the body. Individuals who have undergone stomach surgeries such as gastrectomy, Billroth II gastrectomy, or Roux en Y diversion are at risk of developing beriberi due to changes in thiamine absorption. Less invasive procedures like LSG, gastroplasty, and adjustable gastric banding can lead to mild thiamine deficiency. Neurological signs like confusion, coordination difficulties, and involuntary eye movements may indicate Wernicke's encephalopathy, a severe form of thiamine deficiency. Other neurological issues may involve neuropathy and weakness. These symptoms can manifest weeks to months of weight loss surgery, emphasizing the importance of regular checkups and timely intervention. Additionally, factors such as illness, poor diet, or increased metabolic demands can exacerbate thiamine deficiency, highlighting the necessity for postoperative care. In summary, there is a risk of developing beriberi from thiamine deficiency following stomach resection or bypass surgeries, so vigilant monitoring, early identification, and appropriate treatment are crucial to prevent complications [43,49].

Nearly half of the people who have had Roux en Y surgery might experience thiamine levels without showing any signs, per research findings. A lack of vitamin B1 (thiamine) could emerge around three weeks post surgery in individuals facing vomiting or a considerable decrease in their food consumption. The main worry linked to thiamine deficiency following surgery is the onset of Wernicke encephalopathy [44,47].

Vitamin D

After bariatric surgery, there is a chance of a lack of vitamins, like vitamin D, which may need monitoring. Patients after bariatric surgeries may require vitamin D supplementation because their bodies may not absorb nutrients efficiently. In addition, research showed evidence of a drop in 25 hydroxycholecalciferol (OH) vitamin D (25(OH)D) levels following these surgeries, leading to bone weakening due to reduced calcium absorption in the gut. It is recommended that all individuals undergoing bariatric surgery have their 25(OH)D levels checked before the operation and receive regular blood workups and supplements afterward to prevent deficiencies. Vitamin D and calcium are essential for bone health and bodily functions, and they can be significantly affected by weight reduction interventions [44,47].

Research has found that vitamin D levels may drop by more than one-third from the baseline following stomach removal surgery, which could result in bone loss. Vitamin D deficiency is mainly caused by the procedure and its effects on the body's vitamin D levels. While there is no recommendation for taking vitamin D supplements post esophagectomy, research indicates that doing so might improve bone strength. Supplementing with calcium (1200-1500 mg) and vitamin D (3000 IU) is recommended to decrease bone loss risks resulting from weight changes, diet habits, and nutrient absorption issues caused by surgery. In summary, bariatric surgery and esophagectomy can affect the levels of calcium and vitamin D in the body, which play an important role in sustaining bone strength and overall health [44,47].

Copper

People who have had weight loss surgery, like Roux en bypass or biliopancreatic diversion, may face the risk of experiencing low copper levels because their bodies might not absorb it as effectively as before. Copper deficiency clinical presentation may resemble iron or vitamin B12 deficiencies, presenting challenges in diagnosis. If left untreated, complications from copper deficiency, such as difficulty walking, numbness, tingling sensations, and paralysis, could persist permanently. Healthcare workers typically assess blood levels of copper and ceruloplasmin to diagnose copper deficiency; however, these tests may not always yield results for individuals with obesity-related inflammation. Copper is an element for enzymatic reactions that are crucial for cell metabolism. Specific enzymes like cytochrome c oxidase, superoxide dismutase, and lysyl oxidase rely on copper for function. Copper is important in transporting iron throughout the body, which is a factor in hemoglobin production and other vital proteins. Insufficient levels of copper can result in anemia and immune deficiency due to its effects on white blood cell counts [44,47].

After undergoing surgery or procedures that affect nutrient absorption, it is critical to remain cautious about the potential for developing a copper deficiency. Disregarding this issue could result in blood-related and neurological complications. Monitoring and supplementation are essential to effectively prevent and manage copper deficiency. Copper deficiency may present as blood and nerve disorders, including anemia, neutropenia, and neurological symptoms resembling those of vitamin B12 deficiency. Observing changes in bone marrow analysis can provide insights into how copper deficiency impacts bodily functions. Studies suggest that most copper absorption occurs in the duodenum, with some uptake in the stomach and ileum. The acidity levels in the stomach are crucial for releasing copper from food sources, underlining the importance of maintaining gastric health for optimal copper absorption [45].

It has been reported that Roux-en-Y surgeries can lead to copper deficiency in about half of the cases. Copper is essential for blood production and nerve function and is primarily absorbed in the stomach and at the beginning of the duodenum. Insufficient levels of this micronutrient can cause problems such as low white blood cell count, red blood cell issues, and coordination difficulties. Taking iron supplements alone without copper supplementation may worsen these problems related to copper deficiency [45].

Magnesium

Magnesium is another essential mineral in human homeostasis since it is a cofactor in many enzymatic reactions. Although the actual magnesium circulating concentrations are usually difficult to define, magnesium deficiencies have been reported after bariatric and digestive tract surgery [50]. Important to mention is that the magnesium levels are usually not notable nor severe [45].

Zinc

Zinc is an essential trace element in numerous chemical reactions in human physiology. Around one-third of patients previous to bariatric (BS) surgeries have low zinc levels, probably due to the sequestration of this element in fat tissue. It is important to consider this fact since a drop in fat percentage post-BS may affect zinc bioavailability negatively [42,51]. The same concern arises considering zinc malabsorption after gastrectomy procedures involving the duodenum and jejunum. It is important to mention that zinc levels are sensitive to the inflammation occurring in cancer patients and, therefore, difficult to interpret. Nevertheless, zinc deficiency rates may go even up to 37%. Therefore, zinc supplementation post-BS and gastrectomy procedures are recommended [52].

Proteins

Anatomy changes post-BS procedures cause protein malabsorption, resulting from altered biliary and pancreatic function [40,42]. It is important to mention that protein deficiency does not come only as a malabsorption result, it also comes as a reduced protein intake, especially in meats and dairy products. After bariatric surgery, a minimum of 60 g of protein per day is recommended to maintain favorable albumin levels [51,53,54]. It is recommended to monitor protein intake and albumin presence before and after surgical procedures, together with a balanced diet [55].

Fat-Soluble Vitamins

Vitamins A, D, and K levels are usually affected after bariatric surgical procedures such as BPD, BPD-DS, and long limb RYGB since the jejunum and ileum are key absorption sites for fat-soluble vitamins. The incidence of deficiency is around 50% to 70% at two to four years. Vitamin A deficiency is most frequent in third-world countries and people with liver storage diseases [55]. The clinical manifestation of vitamin A deficiency is more notable and frequent than that of vitamin K [40]. Vitamin E deficiency is rare or only seen years after since, in most patients, there is sufficient liver storage [42,56]. Vitamin A intake is affected by low-calorie restriction and low-fat bioavailability, with the result of low levels of retinol and carotenoids [39]. In case of vitamin A deficiency, 10 mg intramuscularly or subcutaneously for repletion, followed by 1-2 mg/week parenterally or orally after RYGB or BPD for vitamin K is recommended [50].

Preoperative nutritional management for gastrectomy and esophagectomy patients

Cancer is the second leading cause of mortality, following cardiovascular diseases. In the vast majority of patients, esophageal cancer and gastric cancers are linked to malnutrition. This correlation is partly due to the nature of the disease, the tumor's location, and additional factors such as dysphagia, the presence of cancer cachexia, altered metabolism, and tissue wasting [55]. Patients with these cancers commonly endure symptoms related to the tumor, including cancer-related pain, loss of appetite, and difficulties with nutrient absorption. Moreover, attempts to manage the patient's condition may further exacerbate malnutrition. Therapeutic interventions such as surgery, chemo-radiotherapy, or preoperative chemotherapy pose a heightened risk of exacerbating the progressive decline in the nutritional status of patients [57].

In more recent times, there has been an increasing utilization of frailty scores to forecast morbidity and mortality rates, emerging as a significant consideration when evaluating and selecting patients for esophagectomy, particularly in light of the aging population. In fast-track surgery protocols, promptly starting enteral nutrition is crucial to fully leverage the benefits of enhanced recovery. These protocols integrate innovative approaches to anesthesia, nutritional care, pain management, and surgical techniques throughout the preoperative period, all aimed at facilitating patients' postoperative rehabilitation. The nutritional aspects of Enhanced Recovery After Surgery (ERAS) include minimizing preoperative fasting, administering preoperative fluid and carbohydrate loading, and initiating oral intake on the first day following surgery [57]. The use of synbiotics has been shown to prevent postoperative disruption of the intestinal microflora and reduce excessive inflammatory responses, potentially through immunomodulatory effects and inhibition of bacterial translocation. Furthermore, synbiotic administration resulted in a lower incidence of severe diarrhea and reduced interruptions or reductions in enteral nutrition. Patients who received synbiotics also experienced an earlier passage of flatus postoperatively compared to the control group, indicating potential maintenance of intestinal motility [57].

Malnutrition within this patient’s demographic significantly impacts quality of life, diminishes tolerance to chemotherapy, and contributes to reduced survival rates. Effective nutritional management for these patients involves thorough nutritional assessment and support, which could help mitigate the adverse effects associated with malnutrition. Once the assessment is completed, a comprehensive plan for nutritional therapy must be devised. Nutrition goals involve ensuring sufficient nutrient intake to prevent muscle mass loss, regulate inflammation and immune response, improve glucose control, mitigate the hyper-metabolic response to surgery, and provide essential micronutrients and macronutrients to enhance healing and recovery. Supplementation can facilitate a transition to the anabolic state [57]. A study conducted on patients with esophageal cancer revealed a significant increase in resting energy expenditure (REE) levels upon diagnosis. REE plays a vital role in calculating total daily energy expenditure. For individuals with cancer, especially those experiencing heightened inflammation, atypical body composition, or a body mass index (BMI) beyond the normal range, it is recommended to utilize indirect calorimetry for precise determination of individual energy requirements, ensuring accurate assessment [58].

For mildly malnourished patients, preoperative nutrition therapy should be initiated for a duration of seven to 10 days to optimize their nutritional status [7]. However, for individuals at a high risk of malnutrition, it is recommended to undergo a minimum of 10-14 days of nutritional therapy coupled with resistance exercise prior to major surgery [7]. Patients with limited physical reserves, particularly those classified as sarcopenia or frail, may derive greater benefit from a multimodal therapy program spanning four to five weeks, incorporating both nutrition and physical exercise components, given their pronounced synergistic impact on muscle protein synthesis [58].

American Cancer Society's projections for gastric and esophageal cancer in the United States for 2024

Approximately 10,880 deaths from stomach cancer, with 6,490 deaths in men and 4,390 deaths in women are projected. Stomach cancer is expected to account for about 1.5% of all new cancer diagnoses in the United States annually. An approximate 16,130 deaths were attributed to esophageal cancer, including 12,880 deaths in men and 3,250 deaths in women [59]. In recent years, there has been a growing body of research focused on utilizing body composition assessment for diagnosing cancer cachexia and sarcopenia as prognostic indicators in individuals with esophageal cancer [59]. A recent systematic review and meta-analysis, comprising 29 studies involving 3,193 patients diagnosed with esophageal cancer, synthesized the available evidence on various methods for assessing body composition and sarcopenia, aiming to investigate their clinical utility in outcome prediction. The studies included in the review utilized different body composition measurement techniques, with computed tomography being the most common (18 studies), followed by bioelectric impedance analysis (10 studies), and one study employing dual-energy X-ray absorptiometry. Substantial variations were observed in study design, sarcopenia definitions, cut-off points, and muscle measurement techniques utilized across the studies [60].

Malnutrition linked with these cancers has detrimental effects, including heightened rates of postoperative complications, elevated infection risks, delayed wound healing, reduced tolerance to treatment, diminished quality of life, and heightened mortality rates. Malnutrition is a common occurrence in individuals diagnosed with gastric and esophageal tumors, marked by a notable reduction in lean body mass. Performing comprehensive preoperative nutritional assessments is crucial to ensure a precise prognosis and the development of comprehensive treatment plans. Throughout the cancer treatment journey, from diagnosis onwards, it is essential to continuously evaluate the nutritional status of patients. This ongoing assessment aims to understand their nutritional condition and prevent any decline in their overall physical well-being. Furthermore, it helps identify individuals who are undernourished, aiding in the evaluation of surgical risks [61]. A precise diagnosis of malnutrition serves as the foundation for establishing a nutritional treatment plan. Anthropometric measurements, the Patient-Generated Subjective Global Assessment (PG-SGA) tool, body composition analysis, dietary intake assessment, evaluation of nutritional complications, and laboratory tests are commonly utilized to assess the nutritional status of patients before undergoing surgery [61].

A recent meta-analysis has confirmed that patients undergoing oncological gastrectomy or esophagectomy are prone to vitamin deficiencies, particularly in 25-OH vitamin D3. For those at risk of calcium deficiency or osteoporosis, calcium supplementation is recommended, given the heightened risk of vitamin D3 deficiency. Furthermore, it is strongly advised to integrate vitamin D3 supplementation into the preoperative, perioperative, and postoperative protocols for patients undergoing gastric or esophageal cancer surgery. Vitamin B12 supplementation is already recommended after gastric surgery, which is well-supported, and it is also reasonable to consider vitamin B12 supplementation after esophagectomy. Screening patients for vitamin deficiencies before and after surgery is deemed prudent. For practical purposes, current recommendations suggest providing vitamin supplementation similar to post-RYGB guidelines following gastrectomy and SG after esophagectomy [62].

Artificial nutrition is recommended in cases where patients are unable to eat for over a week or if their estimated energy intake falls below 60% of the requirement for more than one to two weeks. Enteral feeding is typically preferred initially, with parenteral nutrition considered if enteral feeding is inadequate or not feasible [48]. According to a recent nonrandomized study, intensive nutritional support provided by dietitians is associated with fewer severe complications following esophagectomy compared to standard care in patients with esophageal cancer (adjusted odds ratio: 0.23, 95% confidence interval: 0.053, 0.97) [58]. While it is widely recognized that a high protein intake is crucial for promoting muscle protein anabolism in cancer patients, determining the optimal amount remains somewhat unclear. Recommendations range from 1.0 g of protein per kilogram of body weight per day to a target range of 1.2-2.0 g of protein per kilogram of body weight per day. Good quality protein derived from sources such as poultry, fish, dairy, eggs, and plants should serve as the primary protein sources, with supplementation only advised when necessary. Encouraging outcomes have been observed with whey protein or essential amino acid supplementation, along with the administration of a bolus containing 20-35 g of protein per meal to stimulate muscle protein synthesis [58].

Postoperative nutritional management after gastrectomy and esophagectomy

There is an improved survivorship among patients following surgery for gastric and esophageal cancers, which necessitates a more thorough evaluation and treatment of postoperative malabsorption. Severe weight loss (>10%) has been identified in a significant number of patients and tends to persist rather than plateau as it does in bariatric surgery. Malabsorptive symptoms are frequently reported post-surgical resection and fat-soluble vitamin deficiencies have been detected until 24 months postoperatively [63]. Some of the identified contributing factors to postoperative malabsorption following esophagectomy and gastrectomy are exocrine pancreatic insufficiency (EPI), small intestinal bacterial overgrowth (SIBO), and bile acid malabsorption (BAM) [63]. EPI is treated with pancreatic enzyme replacement therapy (PERT). SIBO is treated with antibiotics, ideally, rifaximin, whereas BAM is treated with bile acid sequestrants, including colesevelam [64].

Although there is a clear occurrence of micronutrient deficiencies postoperatively, it may not be linked to the surgery itself. Nutritional deficiencies can arise due to anorexia and early satiety, which results in insufficient dietary intake [65]. Furthermore, reduced gastric acid secretion and neoadjuvant therapies also play a vital role in the development of deficiencies [66]. Therefore, oral supplements might be effective in treating common nutritional deficiencies such as vitamin B12, folate, iron, calcium, and vitamin D [65,67]. Several studies suggest that there may be benefits to setting up dedicated clinics to assess postoperative malabsorptive conditions and initiate treatment promptly [63,66,67].

The ERAS protocol suggests initiation of oral or enteral feeding as soon as six to 12 hours post surgery. Patients who underwent gastrectomy for esophageal malignancy are instructed to consume small frequent meals with the avoidance of liquids during meals to treat dumping syndrome. These restrictions are not applicable in the absence of symptoms of dumping syndrome, and patients are simply advised to have small, frequent, low-fat meals [67]. Studies have shown better outcomes in esophageal and gastric cancer patients who received early enteral nutrition (EN) pre- and postoperatively. It is associated with a shorter hospital stay as well as lower mortality [68]. EN poses a lower risk of infectious complications such as pneumonia and septic shock [68]. Data suggests that patients who received seven days of enteral feeds preoperatively had a shorter hospital stay and lower cost of nutrition compared to those receiving parenteral nutrition (PN) [69]. This may be ascribed to the protective effects of EN on the intestinal mucosa as well as improved gastrointestinal tract adaptability. While these studies do suggest a considerable benefit of early initiation of EN and better outcomes compared to PN, there is a requirement for further investigation and multi-center randomized controlled trials [69].

Needle catheter jejunostomy (NCJ) placement may be considered a safe and long-term option to provide nutritional supplementation to patients until six months post upper gastrointestinal tract cancer resection. A retrospective analysis of 102 patients receiving NCJ showed a reduced decline in weight during the first six months following surgery, as well as a decreased loss of body cell mass [70]. Numerous randomized controlled trials highlight the improved outcome in patients on home enteral nutrition (HEN) after esophagectomy. The patients maintained a better nutritional status, body weight, BMI as well as overall quality of life. However, the long-term results of HEN have yet to be analyzed [71,72]. Nutritional management after gastrectomy and esophagectomy needs to be addressed more extensively to improve surgical outcomes and quality of life as well as to promote early discharge. There is also a necessity for a standardized set of guidelines, which includes the frequency of screening tests and the duration of treatment. A multidisciplinary approach, including early dietitian referral to offer nutritional support, can help prevent gastrointestinal complications [73].

Implications for patient care

Consequences for Patient Care

Nutritional deficiencies: Among the most critical consequences of malabsorption and malnourishment following gastrectomy is the onset of nutritional deficiencies. Significant alterations in the composition and operation of the digestive system may lead to inadequate absorption of essential nutrients, including vitamins, minerals, and micronutrients. As a result, patients may experience deficits in important nutrients such as protein, iron, calcium, and vitamin B12. The patient’s general health and well-being may be significantly impacted by these inadequacies [74].

Strategies for Mitigation Techniques

Nutritional modifications: Changing one’s diet is a practical way to prevent malnutrition and malabsorption after a gastric bypass surgery. This method focuses on carefully adjusting the patient’s diet to make up for the altered digestive processes and decreased absorption capacity following surgery. Dietary adjustments are intended to maximize nutrient absorption and minimize gastrointestinal discomfort by modifying the timing and composition of meals [74].

Range of Dietary Adjustments

Developing a schedule of smaller, more frequent meals: It can improve nutrient absorption and reduce gastrointestinal distress by dividing the daily food intake into smaller, more frequent meals [74].

Inclusion of soft and easily digested meals: Including foods that are easy to digest, such as cooked veggies, lean meats, and fully cooked grains, will facilitate better digestion and increase the absorption of nutrients [74].

Supplementation with essential vitamins and minerals: Considering the potential deficiencies that may surface post gastrectomy, it may be necessary to supplement the diet with vitamins and minerals, including iron, calcium, vitamin B12, and folate, to deter nutritional deficiencies [74].

Formulating personalized dietary plans: Teaming up with a registered dietitian to formulate customized dietary plans can facilitate the maximization of nutrient intake while accommodating the patient's unique needs and preferences [74].

Pharmacological Interventions

Another significant strategy involves pharmacological interventions, offering an additional approach to managing malabsorption and malnourishment in gastrectomy patients. These interventions encompass the use of medications or supplements aimed at enhancing nutrient absorption or addressing deficiencies. By leveraging pharmaceutical agents, healthcare providers can target specific aspects of the altered digestive physiology post surgery to optimize nutritional status and improve patient outcomes [75]. Examples of such pharmacological interventions are listed below.

Pancreatic enzyme replacement therapy (PERT): PERT implies the administration of pancreatic enzymes to make up for the diminished enzymatic activity in the digestive system, thus fostering the disintegration and absorption of nutrients [74]. The enzymes come in capsules that are taken with food. These help to digest food by breaking down carbohydrates, fats, and proteins.

Probiotics: Probiotics, like *Lactobacillus* and *Bifidobacterium* species, may contribute to improving gut health and nutrient absorption by reinstating the equilibrium of beneficial gut bacteria [75].

Intravenous nutrient supplementation: In situations where oral intake is inadequate or unfeasible, the intravenous administration of essential nutrients, such as amino acids, vitamins, and minerals, may be required to preserve adequate nutrition [76].

## Conclusions

Our review of the literature helps recognize the importance of understanding the details concerning the main surgeries performed for patient weight loss and eradicating esophageal and gastric cancer. Esophagectomies are performed in malignant and benign scenarios, with a defined criterion for where to perform it. The primary mechanism affected in malnutrition post esophagectomy is the motility of the gastrointestinal system and post gastrectomy, the primary complication is malabsorption of macro and micronutrients; thus, post surgery, the surgical team is encouraged to make sure the patient follows the recommendations regarding dietary modification, vitamin supplementation, and pharmacological interventions. The absorption of nutrients is highly affected post gastrectomy. In the first year, vitamin B12, folate, iron, and vitamin D are difficult to absorb, as well as zinc, copper, calcium, selenium, and vitamins A and B9 find themselves in low concentrations years later. Consequently, patients present anemia, beriberi, bone loss, difficulty walking, paralysis, and low blood cell count. Since both processes of esophagectomy and gastrectomy result in malnutrition, preoperative management focuses on minimizing fasting, administering preoperative fluids and carbohydrates, using symbiotics, and creating a plan of early oral intake post surgery as soon as possible. This management plan may need to be implemented between seven days to five weeks, depending on the initial nutritional state of the patient. Post-surgery management focuses on the ERAS protocol, associating this with shorter hospital stays and lower mortality rates. Analyzing the different factors involving esophagectomies, gastrectomies, and their consequences, we search to highlight the importance of appropriate nutrition and nourishment for patients to experience a better quality of life post surgery, as well as encourage further research, review, and application of the best protocols to ensure better outcomes. We recommend the surgical teams focus on a personalized and holistic approach to the pre-surgery diet, as well as the post-surgery nutrition management, always explaining to the patients the expected changes in their physiology and the necessary habits and medications necessary to live a healthier life.
